# Learning from the Pandemic to Improve Care for Vulnerable Communities: The Perspectives and Recommendations from the Rare Disease Community

**DOI:** 10.5334/ijic.5812

**Published:** 2021-03-04

**Authors:** Raquel Castro, Erwan Berjonneau, Sandra Courbier

**Affiliations:** 1 EURORDIS-Rare Diseases Europe, Paris, France

**Keywords:** integrated care, COVID-19, rare diseases, vulnerable populations, patients, caregivers, remote care

## Abstract

The COVID-19 pandemic has brought about health and social care disruptions for millions of people with complex health needs and disabilities, who are amongst the most vulnerable populations. More than ever before, the need for integrated care is pressing, as fragmented health and care systems struggle to face the pandemic and vulnerable individuals are exposed to aggravated health, social and economic hurdles. The implementation of effective integrated care pathways is long due and must build upon the key guidelines being published by experts worldwide. Furthermore, the pandemic is opening the doors for more remote healthcare and care coordination, with both services and care receivers being increasingly more receptive to virtual and digital solutions, so long as they are fit for purpose and do improve care.

In this article we will go over the impact that the pandemic has had so far for the community of people living with a rare disease, and how it has aggravated their already vulnerable condition. We will also recall crucial recommendations to achieve integrated care for those with complex needs and will bring to light the most recent perspectives of the rare disease community in regards to remote care. While we are still facing the COVID-19 crisis, we do hope that by 2030 we will look back at this pandemic as the ultimate propeller which led millions of people with vulnerable conditions to finally be supported by effective integrated and holistic care pathways.

## COVID-19 generated significant care disruptions for people living with a rare disease

Today, people living with a rare disease and their family members remain a marginalised and largely invisible population, with little information about their diseases and their rights, few treatments, and a high level of health, psychological, social and economic vulnerability [1]. Integrated care, within the health system, and between health, social and community services is essential for people with rare diseases – which are often chronic, highly complex and severely disabling.

A large scale European survey – ‘Juggling care and daily life’ (2017) – has demonstrated many of the care coordination challenges faced by people with rare diseases: 65% of respondents had to visit different health, social and local services in short periods of time and 67% found that these services communicate badly with each other. As a consequence, 6 in 10 found their care hard to manage: “Inter-professional communication works only through the good intentions and efforts of particular individuals. Departments communicate with each other primarily through patients or their parents. This is one of the main difficulties in the lives of the families”, stated a survey participant from the Czech Republic. Due to their disease and to the fragmented care pathways, a striking number of 7 in 10 respondents had to reduce/stop their professional activity [2].

The COVID-19 pandemic has exacerbated these challenges. During the first wave of crisis in Europe, access to the health care, social services and treatment was disrupted, and the stress and anxiety of daily life heightened, as demonstrated by the large scale survey to the rare disease community ‘How has COVID-19 impacted people with rare diseases?’ (2020) [3,4]. A respondent from Belgium commented: “We no longer have a follow-up concerning the transplant. Psychological and psychiatric follow-ups have been cancelled and private care is too expensive. I have a hyperactive son in the house 24 hours a day”.

By the end of May 2020, 8 in 10 people with a rare disease in Europe had already faced care disruptions, with 6 in 10 finding them detrimental to the person’s health, while 3 in 10 considered that these could probably (21%) or definitely (9%) be life-threatening. Access to social and support services was also disrupted, with at-home support and personal assistance being reduced, leaving many people with limited essential daily-life support [5].

Centres offering rehabilitation therapies, physiotherapy, respite care and day care had to close or to reduce their services. Several of these rapidly sought to convert part of their services to virtual delivery, initially facing low receptivity from beneficiaries and public/private funders [5].

The impact of the pandemic on the rare disease community spanned across access to diagnosis, health care, treatments and social services. Their employment, education and mental health were also severely affected [5]. “Suddenly there was no longer much help or contact. Some operations that should have been done have been postponed. I’m in a lot of pain. In addition, I have the feeling that I am on my own.”, shared a survey respondent. Never before has the need for integrated care been this pressing.

## Health care and welfare systems must become more integrated and resilient

To ensure that the most vulnerable populations receive the support they need, national health and welfare systems need to become more coordinated and resilient, ensuring integrated care at all times and even more during a crisis.

These recommendations, developed by the rare disease community and wide group of stakeholders, are crucial to ensure integrated care to the most vulnerable [1,6]:

- Entitling all people to an individual, person-centred care plan, delivered within a multidisciplinary, holistic approach in coordination between all care providers;
- Guaranteeing the coordination and interoperability between national policy sectors and between service providers via e.g. Inter-Ministerial working groups, shared budgets and case management services [7];
- Empowering people with lived experience and meaningfully engaging them in the design and delivery of policies and services;
- Supporting networks and services which collect and disseminate data and good practices.

These recommendations are reinforced by the ongoing multi-stakeholder foresight study for rare diseases, which proposes as the ideal scenario of better care for people with rare diseases in Europe a society where, by 2030, healthcare and welfare systems are led by holistic needs and integrated care plays a crucial role [8].

## Remote health care and virtual case management bring important value, but are not fit for all purposes

The COVID-19 pandemic has certainly influenced the relationship of people with rare diseases and others with complex conditions with the healthcare and welfare systems, opening doors to more digital health options.

Remote health services, such as virtual/phone consultations and electronic prescriptions are playing a crucial role during the crisis. Amongst the respondents to the European survey on the impact of COVID on the rare disease community, by the end of May 2020, 52% had used online/remote consultations since the start of the pandemic, 47% had received a prescription via email and 21% had participated in online training to manage their disease. 9 in 10 respondents found these experiences useful [3,4]. “The doctors called me and then sent me the prescription to save me the waiting time in the consulting room. I thought that was very good”, said a survey respondent.

When people already know their health care team, remote care can help compensate care disruptions and improve overall access to care: “My rheumatologist phoned me to check on my progress since discharge from hospital and adjusted my prescription based on this”, stated a person with a rare disease from the United Kingdom. Remote care can also be used to optimise the time and travel expenses associated to care: “Before each planned consultation, there is a telephone conversation and together we clarify whether a consultation on site is necessary”, shared a person with a rare disease in Switzerland.

Many people with a rare disease who responded to the survey consider that remote consultations could be part of their routine care. A respondent from Denmark commented: “My semi-annual check was changed to a telephone consultation instead, which could easily be the case for every other check in the future”. Discussing results of a routine medical analysis or adjusting a prescription are also considered particularly appropriate remote consultations: “I am currently in a good shape and it is enough for me to speak to my family doctor by phone to discuss a blood test”, said a person with a rare disease from Germany.

The lessons learned from the pandemic should serve to encourage more virtual care in the future, not only via remote consultations and e-prescriptions, but also with the provision of virtual case management or certain follow-up rehabilitation/therapy sessions. As such, health and social services which are seeking to convert part of their services into quality remote services must continue to receive adequate funding.

Despite its benefits, remote care should only be used under specific circumstances. For the rare disease community, nothing can replace certain procedures and the human contact – in particular when meeting a new care professional, when receiving a diagnosis, when sensitive or highly impactful information is announced, or when important decisions on their care are to be made. A survey participant from France commented: “A consultation by phone is better than nothing but this is not ideal to discuss the idea of a transplant.”

## Conclusion

COVID-19’s impact on people with complex conditions was aggravated by the deficient coordination and integration between health, social and community services. People living with a rare disease faced significant care disruptions, which affected their health and wellbeing. To compensate the care disruptions, they quickly adjusted their care expectations and experiences, adhering to remote care, which many services rapidly started to offer. The provision of remote care, by the regular care teams, has been appreciated as a means to guarantee the continuity of care while also saving time. However, remote care will not be the solution to all the current care gaps. Integrated care, provided in a person-centred and holistic manner, remains an urgent priority to be addressed in order to ensure the wellbeing of the most vulnerable health populations within this decade.
